# Advancing Rural Health Through Nursing Education, Community Partnerships, Research, and Workforce Development

**DOI:** 10.3390/healthcare14213767

**Published:** 2026-11-04

**Authors:** Lea Niederbaumer, Michelle Lichtenberg, Danielle M. Schievelbein, Abby O. Seydel, Nicole Carlson, Theresa Garren-Grubbs, Danielle Currier, Brian Williamson, Amanda Mehlhaff, Marie Schmit, Sarah Mollman

**Affiliations:** College of Nursing, South Dakota State University, 1015 Campanile Avenue, Brookings, SD 57007, USA; lea.niederbaumer@sdstate.edu (L.N.); danielle.schievelbein@sdstate.edu (D.M.S.); abby.seydel@sdstate.edu (A.O.S.); nicole.carlson@sdstate.edu (N.C.); theresa.garrengrubbs@sdstate.edu (T.G.-G.); danielle.currier@sdstate.edu (D.C.); blwilliamson@jacks.sdstate.edu (B.W.); amanda.mehlhaff@sdstate.edu (A.M.); marie.schmit@sdstate.edu (M.S.); sarah.mollman@sdstate.edu (S.M.)

**Keywords:** rural health, nursing education, workforce development, frontier health, rural nursing, health disparities, academic-practice partnerships, land-grant university, community partnered research

## Abstract

**Highlights:**

**What are the main findings?**

- A rural, land-grant college of nursing can address rural health challenges through innovative educational pathways, workforce development programs, academic–practice partnerships, and community-engaged research.
- Targeted initiatives, including rural clinical training, online “train-in-place” nursing education, Native American student support programs, and research addressing workforce well-being and workplace violence, may contribute to preparation and retention of nurses in rural and underserved communities.

**What are the implications of the main findings?**

- Rural nursing workforce challenges require integrated solutions that combine education, community partnerships, workforce support, and research rather than isolated interventions.
- The strategies implemented by the South Dakota State University College of Nursing highlight approaches that may be replicable for other rural and frontier regions seeking to strengthen healthcare access, workforce capacity, and community health outcomes.

**Abstract:**

Background: Rural populations experience healthcare disparities, including workforce shortages, geographic isolation, limited access to specialty services, and adverse social determinants of health. These challenges are particularly evident in South Dakota, where nearly half of the population resides in rural or frontier communities. Because of these challenges, preparing nurses to work in rural America is critical to building a workforce capable of meeting this population’s unique healthcare needs. Methods: This article is an experience-based institutional case essay describing an integrated approach to how South Dakota State University College of Nursing, located within a rural land-grant institution, has developed and adapted educational programs, workforce initiatives, community partnerships, and research programs to address rural health challenges across the state and region. The College of Nursing (CON) includes three campus locations—Brookings, Sioux Falls, and Rapid City—and includes undergraduate and graduate nursing education. Nursing education is designed to allow students to blend their nursing education with South Dakota’s unique rural environment. Results: The CON offers multiple pathways into the profession, including traditional and accelerated Bachelor of Science in Nursing (BSN) programs, an online BSN option utilizing a train-in-place model, master’s-level nursing education and administrator programs, Doctor of Nursing Practice (DNP) programs, and PhD education. Through strategic academic–practice partnerships and federally funded workforce initiatives, the college prepares nurses for rural practice across primary care, acute care, long-term care, and underserved settings. Additional initiatives support Native American student success, rural workforce recruitment and retention, oral health outreach, care coordination for children and youth with special healthcare needs, and research focused on workforce well-being, workplace violence, and healthcare access. Conclusions: A multi-pronged approach by rural colleges of nursing may improve rural healthcare access, strengthen the nursing workforce, and address the complex health needs of rural and frontier populations. South Dakota State University CON demonstrates how innovative educational delivery models, community partnerships, targeted workforce initiatives, and research can contribute to sustainable rural healthcare solutions and rural health.

## 1. Introduction

The United States experienced improved health outcomes and lower mortality rates in the first two decades of the 21st century; however, when comparing rural areas with urban/metropolitan areas, health disparities become apparent and are well-established. In fact, the gap between urban and rural mortality rates tripled between 1999 and 2019 [1]. Health disparities in rural areas are attributed to numerous barriers to rural residents accessing quality healthcare, including healthcare workforce shortages, greater distances to healthcare services exacerbated by a lack of reliable transportation, and a lack of broadband internet with fast enough speeds to support telehealth [2]. Furthermore, rural counties have worse social determinants of health than urban areas [3], such as higher rates of uninsured residents, higher poverty rates, lower educational levels [2], lower average incomes, and fewer available jobs [4]. These factors contribute to rural residents having lower health literacy rates than their urban counterparts, leading to reluctance to seek healthcare, difficulty understanding how to navigate the healthcare system, and challenges in communicating with healthcare professionals [2].

On average, 20% of the United States population resides in a rural area [5] experiencing these barriers to accessing quality healthcare. In South Dakota (SD), nearly half of the state’s approximately 1 million residents live in rural areas. SD ranks eighth in the nation in highest percentage of rural residents and eighth in lowest population density [6], meaning that the approximately 500,000 rural residents are geographically distant from each other, a community, and healthcare services. In fact, a recent study found that rural cancer care patients in SD travel 60–300 miles round trip to access care. This is compounded by other transportation considerations, such as weather and having a reliable vehicle, driver, and money to travel these distances [7].

As the most trusted, meaning ethical and honest, profession in the nation for two and a half decades [8], nursing has the position and ability to improve access and quality of rural healthcare [9]. Since 1862, land-grant universities have had the mission and responsibility to bring higher education and services to the state’s residents while also addressing the needs of communities. One of the challenges in the 21st century for land-grant universities is meeting rural community needs in the context of a predominantly urban America [10]. Hence, colleges or schools of nursing at land-grant universities, especially those in rural states, are well positioned to contribute to efforts to address rural healthcare challenges. This paper describes a multi-pronged approach undertaken by a college of nursing at a state land-grant university to meet the needs of rural communities and adapt to changes in the rural healthcare landscape.

### 1.1. Defining Rural

Rural is defined using the Health Resources & Services Administration (HRSA) definition, which considers, in part, non-metropolitan counties and primary Rural-Urban Commuting Area (RUCA) codes 4–10 [5]. In the state of SD, located in the upper Midwest of the United States, the majority of the 66 counties are classified as rural (63 counties). Of these 63 rural counties, 34 are considered frontier, defined as fewer than six people per square mile [11]. In a state with such significant rurality and approximately half of the population living in these rural and frontier areas [12], a multi-pronged approach is needed to meet the needs of rural communities and an ever-changing rural healthcare landscape.

### 1.2. Rural Healthcare Access and Quality

The healthcare landscape in SD includes 39 critical access hospitals, one rural emergency hospital, 57 rural health clinics, and 38 Federally Qualified Health Centers [13]. The state experiences significant health professional shortages, with most of the state designated as medically underserved areas (97%) and/or health professional shortage areas (88%), and with the entire state being designated as mental health professional shortage areas [14].

The healthcare workforce shortage in rural SD is exacerbated by the decreasing working-age population in rural areas, leaving older residents without access to healthcare services. Additionally, as the rural population decreases, they are more likely to be geographically isolated from others, including family, friends, communities, and healthcare professionals [7]. While access to healthcare and isolation vastly affect rural residents, healthcare professionals also lack access to other healthcare professionals for consultation and often practice in isolation [7].

### 1.3. Social Determinants of Health

Any approach to addressing rural healthcare challenges must consider the social determinants of health (SDOH) that impact the healthcare received by rural residents. Those living in rural areas experience less access to resources, incomes lower than their urban counterparts, and lower education levels [15]. In SD, the 2024 poverty rate was 7.5% in urban areas compared to 13.8% in rural areas [16]. Additionally, the state is home to nine federally recognized American Indian tribes who experience severe economic disparities, including substantially higher poverty rates of 22–48% [11] and unemployment rates as high as 89% [17]. This is a vast difference from the state average poverty rate of 10% [11] and unemployment rate of 2% [18].

## 2. Rural Workforce Supply

The supply of all health professionals except Licensed Practical Nurses (LPNs) in rural areas is lower per 10,000 population than in urban areas [19]. For example, compared to urban settings, which have 31.2 physicians per 10,000 people, rural areas have a mere 13.1 physicians per 10,000 people [20]. Additionally, more than half of rural physicians aged 50 and older are projected to retire by 2030, adding to a 23 percent decline in the rural workforce. This projected shortage exemplifies the existing and long-standing challenges of delayed care and increased travel distances for the rural population [20]. The growing shortage of rural physicians not only limits timely access to medical care but also increases reliance on the rural nursing workforce to meet the healthcare needs of underserved populations. As physician shortages intensify and rural populations continue to age, nurses are increasingly called upon to fill critical gaps in care delivery, making the stability of the rural nursing workforce essential to rural health outcomes.

Nationally, the rural nursing workforce is older, less diverse, less educated, and receives a lower salary than their urban counterparts [21]. This creates a looming rural nursing workforce crisis as older, more experienced nurses retire [21] and the population ages and requires more healthcare [22]. Projections by the Bureau of Labor Statistics indicate almost 200,000 RN openings annually through 2034. The number of openings is attributed to nurses transitioning to other careers or exiting the workforce through retirement [23]. According to the SD Department of Labor and Regulation [24], there are projected to be 1028 registered nurse (RN) openings annually through 2032, representing a 13.2% increase in total employment for RNs in the state. According to Nursing Education [25], SD will rank third in the USA for a nursing workforce shortage by 2030, with a projected shortage of 14%.

An often-forgotten workforce in healthcare is nursing assistants (NAs), who provide a significant amount of direct care, especially in long-term care (LTC). NAs help with activities of daily living such as bathing, dressing, toileting, eating, and ambulating [26]. NAs also assist with transferring, repositioning, taking vital signs, and serving as a liaison between the resident and their nurse [27]. Although the terms certified nursing assistant (CNA) and NA are often used interchangeably, a CNA is a NA who obtains state certification through a training program and exam. The specific requirements vary by state [28]. We will use the broad term of NA, which includes certified and non-certified NAs.

Most NAs within the United States do not have any post-secondary education, are female, and are people of color. Furthermore, NAs earn low incomes [29]. The rural setting includes additional unique challenges for NAs, including a risk of health disparities related to lower socioeconomic status, as well as decreased healthcare specialist access [30]. Rural areas have fewer NAs available to care for older adults compared to urban areas, as indicated by a lower NA ratio to the older adult population [31], further exacerbating the staffing crisis in rural areas. The NA shortage has been attributed to low pay and recruitment difficulties. Furthermore, the COVID-19 pandemic worsened already high turnover rates [32].

### 2.1. Rural Nursing Workforce Solutions

Consistent with the university’s land-grant mission, the College of Nursing (CON) plays a vital role in advancing opportunities for the health and well-being of individuals and communities across SD, the region, and the nation [33]. As a leader in nursing education, the CON graduates nearly half of the state’s nurses prepared at the Bachelor of Science level (BSN) and has a longstanding history of adapting its programs to meet evolving healthcare workforce needs. In response to SD’s nursing workforce needs, particularly in rural and medically underserved areas, the CON has implemented multiple strategies to expand access to nursing education and address identified workforce gaps. These efforts include graduate programs aligned with state healthcare needs, such as psychiatric/mental health offerings for BSN- and master’s-prepared nurses, as well as refresher courses for RNs and LPNs seeking professional recertification. Further, the CON offers a pathway for nurses prepared at the associate degree level (ADN) to earn a bachelor’s degree through the RN to BSN program. These programs demonstrate the CON’s continued commitment to expanding access to nursing education across the state.

Most recently, CON launched online delivery of its BSN program in spring 2026. The five-semester BSN program has traditionally been offered through standard (2.5-year) and accelerated (1-year) options across three geographically dispersed locations, with two in eastern SD and one in western SD, approximately 390 miles from the main campus. The online option was developed primarily to serve students who live at a distance from existing CON sites and who may be unable to relocate or regularly travel to participate in a campus-based program. With capacity to admit up to 24 students annually, the program provides an additional pathway to prelicensure nursing education while allowing students to remain primarily in their home communities. Designed as a “*train-in-place*” model, the program uses innovative online and distance-learning technologies to allow a substantial portion of students’ didactic, lab, and simulation learning to be completed remotely, providing flexibility for students balancing education with work, family, agricultural, and community responsibilities.

The online BSN program was developed to promote consistent competency development through a standardized blend of in-person experiences, innovative technology-enhanced learning, and community-based clinical education. Each semester, students attend immersion experiences at the closest of the CON’s three sites to complete specialty nursing clinicals, high-fidelity simulations, and skills that require direct faculty supervision and hands-on competency validation. Advancements in virtual reality, distance simulation, and other educational technologies allow students to complete additional skills practice, assessments, and simulated clinical experiences remotely while remaining in their home communities. Students also complete approximately 400 of the 480 required clinical hours off campus through partnerships with rural and community healthcare organizations, providing direct patient-care experiences close to home. Together, these approaches are intended to support consistent competency development while reducing travel demands and strengthening partnerships with rural and community healthcare organizations. Because the online BSN option was launched in 2026 and the first cohort has not yet completed the five-semester program, completion, rural employment, and rural workforce retention outcomes are not yet available. These outcomes will require longitudinal evaluation as cohorts progress through the program and enter nursing practice.

The online BSN program represents one component of the CON’s broader efforts to strengthen the rural nursing workforce through education, workforce development, and community partnership. These efforts build upon the CON’s longstanding history of securing federal funding through the Health Resources and Services Administration (HRSA) to support the recruitment and retention of nurses in rural communities. Recruitment focuses on attracting healthcare professionals to current and future workforce opportunities, while retention focuses on sustaining that workforce within rural facilities and communities [34]. Innovative approaches that address both recruitment and retention are essential to strengthening the rural nursing workforce. Both the SD Department of Health [14] and the World Health Organization’s (WHO) Workforce 2030 strategy [35] identify education and workforce training as important strategies for addressing healthcare workforce shortages. Several of the CON’s approaches to addressing the rural nursing workforce shortage will be described. While many are supported through federal funding, the CON has made intentional efforts to sustain those with positive impacts.

#### 2.1.1. Rural Nursing Solution—Native American Nursing Education Center

The CON demonstrates a commitment to meeting the needs of rural healthcare through the Native American Nursing Education Center (NANEC). NANEC’s mission is to provide a nurturing and collaborative environment and aims to promote recruitment and retention among Native American students by providing financial, academic, social, and cultural services. The center provides comprehensive support to students in nursing programs, including childcare, food, gas, tutoring, traditional ceremonies, and mentorship from Lakota elder nurses. NANEC mentors approximately 30 students each year who participate in pre-nursing, standard, accelerated, RN-BSN, and doctorate programs. NANEC’s comprehensive student support has had remarkable success increasing BSN graduates per year from an average of 3.15 (2015–2020) to 7.6 (2020–2026), including an 83% retention rate (66 graduates out of 80 enrolled students from 2015–2026) and 100% NCLEX pass rate (66 of 66 from 2015–2026). While other institutional and college strategies may have affected these positive outcomes, graduates consistently report that NANEC was the main factor in their success. Across all nursing programs, there have been 66 graduates (2015–2026), with 59 (89%) practicing in SD and 36 (55%) practicing in Native American healthcare facilities within 2–9 months of graduation. Most NANEC graduates are practicing full-time to fulfill their service obligation for federal financial assistance (scholarships during the program).

#### 2.1.2. Rural Nursing Solution—Rural Fellowship

The Rural Nurse Fellow Program offered by the SDSU CON provides an opportunity for senior nursing students to complete a 144 h preceptorship experience (capstone/directed study) in a rural healthcare facility. The program is designed to provide exposure to rural healthcare, to understand issues facing rural healthcare providers, and to allow nursing students to develop the specific skills necessary for practicing in rural hospitals. Following completion of the program, students are designated as a Rural Nurse Fellow. Since the program began in 2013, over 100 students from the CON have participated. Post-graduation employment data is not available for this program. The fellowship’s timing (the last semester of the BSN program) is well-positioned to affect students’ employment plans; therefore, the college has sustained it as an ongoing opportunity for senior BSN students.

#### 2.1.3. Rural Nursing Solution—Primary Care

Supported by a HRSA grant, Impacting Models of Practice and Clinical Training for Registered Nurses and Students (IMPACT-RNS) was implemented in 2018. The goal of the project was to increase nursing student clinical training in rural primary care to increase rural nurse education and practice, increase quality and retention, and recruit students to serve as RNs in rural primary care post-graduation. Since grant funding ended in 2023, the project has continued and is now funded by CON donors. IMPACT-RNS is open to all university nursing students who have a desire to learn more about rural primary care and underserved populations. Students complete 48 clinical hours during their fourth semester and 54 clinical hours during their fifth semester. Over 90 students from the CON have participated in this program. While donor funding remains available to support this program, many primary care staffing structures have shifted away from the utilization of RNs. Responding to this shift, this funding will support other rural nursing experiences, such as the rural nurse fellow program, in the future.

#### 2.1.4. Rural Nursing Solution—Acute Care

PREPARE-RNS, Partnering to Address the Critical Nursing Shortage in South Dakota, is a part of the Nurse Education Practice Quality and Retention—Registered Nurse Training Program (NEPQR-RNTP) through HRSA. Awarded through HRSA, this grant provided funding over a total of four years (2022–2026). This multi-year program sought to prepare pre-licensure BSN students for careers in rural acute care settings through the creation of experiential learning opportunities and training models, which provided education on topics such as the SDOH, health equity, and health literacy. Students interested in working in rural and/or underserved communities were prioritized for acceptance into this program.

This was an academic–clinical partnership between this land-grant institution and a regional health system with a large rural footprint. Pre-licensure students were selected through an application process to participate in this program to identify students’ current understanding and experiences in rural acute care, along with their desire to work in a rural area post-graduation. Previous studies have identified that preferential selection of students from rural backgrounds, along with training in rural areas, increases retention of health professionals in rural areas [36]. This program was modeled after similar rural training track programs in medical education, which support the idea that medical students who are exposed to rural training opportunities are more likely to practice in a rural area compared to students who do not participate in these experiences [37,38,39]. While evidence from rural medical education informed program development, differences between medical and nursing education should be considered when interpreting applicability of these findings. Prior to this award, most nursing workforce recruitment and retention strategies in the published literature focused on scope of practice adjustments, loan repayment programs, and train-in-place models [40].

A total of four cohorts were recruited for this program, with eight students being accepted for each cohort. Students in this program completed over 80 h of clinical in a rural acute care facility, followed by a 144 h 1:1 experience with a nurse preceptor in a rural acute care facility during the last year of the nursing program, providing students with a unique look into rural practice. In addition to clinical experiences, PREPARE-RNS students participated in an ECHO Professional Development model, completing over 10 h of education and training with subject matter experts on the SDOH, health equity, and health literacy. It is modeled after similar rural training track programs in medical education, which support the idea that medical students exposed to rural training opportunities are more likely to practice in a rural area compared to students who do not participate in these experiences [37,38,39]. Students selected for this program received tuition and travel support, as many rural experiences required greater travel distances than traditional clinical opportunities offered within the nursing program.

Grant funding for this program ended in Fall 2026. In total, 43 qualified applicants applied over the course of the program. A total of 33 students participated in the program, with 31 (93.9%) students completing the program in its entirety. One student completed only the first semester, and another completed only the second semester. Although preliminary workforce outcomes indicate more than half of the participants accepted employment in rural facilities following graduation, final workforce outcome data are currently undergoing analysis. While this program supported experiential learning opportunities in rural acute care, as well as education on the SDOH, health equity, and health literacy, students with an interest in rural health and practicing in a rural area post-graduation may have been disproportionately represented in the sample. However, because the purpose of this program was to recruit and retain nurses in rural practice settings, prioritizing students interested in rural nursing was an intentional component of the program design.

This program did face challenges throughout implementation. While an agreement was in place between the critical access hospital and the university for clinicals to be completed during the first semester of the program, several nursing schools utilized the hospital for clinical experiences. The size of the hospital did not have the ability to support more than one group of nursing students at a time. Therefore, the hospital and university worked together to identify the best clinical days each semester to meet the needs of both entities. As this grant has now ended, the lessons learned from this program were addressed in RETAIN-RNS (described below) to strengthen the program moving forward.

#### 2.1.5. Rural Nursing Solutions—Acute and Long-Term Care

RETAIN-RNS, Partnering to Expand and Retain South Dakota’s Nursing Workforce in Acute and Long-Term Care Settings, is a part of the HRSA Nurse Education Practice Quality and Retention—Workforce Expansion Program. This award is over four years (2025–2029). This program is still in progress, with only the first year having been completed. This multi-year program seeks to prepare pre-licensure BSN students for careers in rural acute and long-term care settings and continues the academic–clinical partnership developed through PREPARE-RNS. Specifically, this grant seeks to expand the nursing workforce by increasing the number of RNs prepared to practice in rural acute care and long-term care settings, as well as enhance nursing education and training with didactic and experiential learning opportunities emphasizing rural practice, health equity, and cultural competence. An additional aim is to strengthen clinical training infrastructure by developing and supporting nurse preceptors, clinical faculty, and sustainable academic–practice partnerships. Modeled after PREPARE-RNS, this program includes over 84 clinical hours in rural acute care followed by a 144 h 1:1 preceptor experience in a rural acute setting. In addition, students complete a summer clinical immersion program, which includes 40 h of training in rural long-term care and 40 h of training in rural acute care. The summer experiences focus on students working closely with BSN-prepared nurses in rural settings to gain an understanding of the specific roles of BSN-prepared nurses. Students also participate in five Project ECHO sessions focused on cultural competency, SDOH, health equity, policy and advocacy, and sustainable solutions and community assets, which include discussion on community health workers as a key member of the healthcare team. In total, this program will consist of five cohorts of students, with no more than eight students per cohort. The first cohort, consisting of eight students, began the program in Summer 2026. This program is projected to support 40 students total.

##### Rural Nursing Solutions—Acute and Long-Term Care

Growing Underserved Communities through Innovation, Development, and Education for Registered Nurses and Students (GUIDE-RNS) is a 4-year grant program (2025–2029) funded through the Health Resources and Services Administration (HRSA) Nurse Education Practice Quality and Retention—Workforce Expansion Program. The program is completing its first year and aims to develop and deploy a training program for BSN nursing students, expand academic–practice partnerships, and collaborate with practice partners to develop, implement, and sustain innovative teaching strategies. The program focuses on preparing students to care for individuals in rural and underserved communities in acute and long-term care settings. The program provides didactic instruction to all nursing students across the program, including information related to distance simulation, SDOH, and health equity. This didactic information provides all students with insight into what to consider when caring for patients in rural or underserved settings. SD is largely rural; therefore, this information is crucial for all students planning to stay here, as they will care for individuals from these settings.

Students in the GUIDE-RNS program receive 80 additional hours of clinical training, comprising screen-based, virtual, and hands-on simulation experiences, as well as clinical experiences in rural or underserved acute and long-term care settings. Students complete these additional hours across three semesters in the nursing program. Of the 80 h, 48 h are spent in the clinical setting, while 32 h are spent completing simulation education. The hours in the clinical setting are split between acute and long-term care, with students completing 24 h in each. A total of 46.5 h is spent in the long-term care setting between simulation education and the clinical setting. Students are 1:1 with a preceptor during their time in the clinical setting, allowing them to work with individuals from rural and underserved communities. This allows students to gain unique perspectives on caring for individuals while working in these environments. Education is also provided to current RNs as part of their preceptor role for the students through Nursing Continuing Professional Development (NCPD) activities. These NCPD opportunities include distance simulation, SDOH, health equity, and facilitating students. The NCPD opportunities will be made available to all nurses across SD once the first round of preceptors has been trained. Currently, 20 students have been enrolled in the program, with plans to admit an additional 120 over the next 3 years, for a projected total of 140 students completing the program. The first cohort of eight students will graduate in December 2026.

### 2.2. Rural Nursing Workforce Solutions—Summary

The NANEC (*n* = 66), Rural Nurse Fellow program (*n* = 100), and IMPACT-RNS (*n* = 90) efforts have been designed, implemented, and sustained by the CON, resulting in 290 nursing students being trained in rural healthcare. PREPARE-RNS trained an additional 33 students and will transition into the RETAIN-RNS effort, which intends to train 40 students. As GUIDE-RNS is implemented, we project another 140 students will gain experience in rural healthcare. This equates to nearly 500 unique nursing students being trained for rural practice between 2013 and 2030; see Table 1. Each program collects enrollment data, and the authors verified that no students are enrolled in more than one program, preventing duplicate enrollment reporting. NANEC and PREPARE-RNS have data indicating that these students are likely to practice in rural settings.

As a land-grant institution, the mission of SDSU is to offer “a rich academic experience through inspired, student-centered education, creative activities and research, innovation and engagement that enhances the quality of life in South Dakota, the region, the nation, and the world” [41]. These opportunities for nursing students reflect the university’s mission while also supporting rural workforce development in a predominantly rural state. These experiences help to develop a nursing workforce prepared to address the ever-changing healthcare landscape for those residing in rural and underserved communities.

## 3. Environmental and Occupational Health Challenges

Burnout among healthcare professionals remains a critical threat to workforce stability, quality of care, and health system sustainability, with nearly half of clinicians reporting frequent burnout and an increasing proportion considering leaving the profession [42]. Burnout is associated with impaired clinical decision-making, reduced communication effectiveness, and diminished capacity, placing both healthcare workers and patients at risk [43]. These challenges are amplified in rural settings, where healthcare workers face unique occupational stressors, including workforce shortages, broad scope-of-practice demands, limited specialty support, scarce resources, and rapidly evolving technologies without proportional infrastructure or training [44]. Hence, the CON is focusing research on combating burnout as a strategy to improve rural nursing workforce retention.

### 3.1. Workforce Well-Being and Burnout

Researchers have traditionally measured the effects of acute stress on worker well-being using self-reported measures, questionnaire-based assessments, and periodic monitoring of biometric and biochemical markers over longer time frames [45]. While informative, these approaches are inherently reactive and do not capture the dynamic physiologic stress and physical fatigue experienced during real-world clinical work. This limitation is particularly consequential in rural healthcare settings, where unanticipated workload fluctuations and limited staffing create sustained physiologic demands that traditional assessments may not fully capture. Addressing the underlying causes of burnout through proactive organizational interventions is essential to minimizing its impact on patient care [44]. Wearable biosensors provide a promising approach to address this gap by enabling continuous, real-time measurement of physiologic stress responses and physical exertion during clinical practice [Barac et al.]. The real-time physiological responses that wearable biosensors can measure include heart rate, blood pressure, body temperature, movement, and cognitive load to diagnose, monitor, and manage health conditions [46,47].

Prior biosensor research has shown that wearable technologies can detect physiologic markers associated with stress, fatigue, and occupational well-being. Twenty-four percent of the orthopedic faculty and resident surgeons had a negatively trending heart rate variability (HRV) as their clinical rotation progressed, indicating increasing physiologic strain and burnout risk as measured by the self-reported Maslach Burnout Inventory (MBI) [48]. A systematic review concluded that 11 of the 17 studies had statistically significant findings on HRV responses to staged stress-generating situations [49]. Huang and colleagues [50] found a statistically significant relationship between participants’ mental and physical fatigue and their heart rate (HR), HRV, and intervals between successive heartbeats (RR intervals), which are all commonly used in physiologic monitoring. However, fatigue experienced by each participant completing a fatigue-inducing quiz may differ from that in the natural work environment. A scoping review of 74 studies concluded that electrodermal activity (EDA) wearables can accurately predict perceived stress or stress-related behavior with an average accuracy of 82.6% [51]. Despite these findings, the comparability of the studies is limited given the variations in EDA processing methods and the perceived stress measurement used [51].

Despite the growing use of wearable technology, few studies have explored the association between physiological responses and burnout, depression, stress, or anxiety [52]. Furthermore, a lack of studies in rural healthcare environments measuring burnout, depressive symptoms, stress, or anxiety among healthcare workers exists. Members of the CON have sought to add to the research in this area by testing wearable biosensors on nursing students in a simulated environment. Wearing biosensors in this environment allows investigators to test their acceptability and feasibility for performing clinical skills while considering patient privacy and hygiene best practices, with future research planned for healthcare workers in rural practice settings.

#### 3.1.1. Nursing Student Well-Being During Simulation

To prepare for future research aimed at better understanding the stress and fatigue of the rural healthcare workforce, which may contribute to their overall well-being and burnout risk, members of the CON conducted a pilot study on nursing students in a high-fidelity simulation environment. The main aim of this study was to explore whether wearable physiologic sensors can unobtrusively capture meaningful stress-related signals in healthcare student contexts and support the extension of this approach to real-world rural clinical environments [53]. A secondary aim was to investigate the fatigue and cognitive load nursing students experienced during the simulation. The biosensors measured ECG (electrocardiogram) and GSR (galvanic skin response) to detect HRV and electrodermal activity (EDA). The study found increased GSR peaks and higher heart rate variability at key simulation points, including task changes and the start and end of the simulation. These findings suggest that simulation demands may contribute to cognitive load or fatigue. They also offer insight into the stress and fatigue healthcare workers may experience in practice, which may affect patient safety, well-being, and burnout. Investigators determined that the wearable biosensors used in this study are not acceptable for healthcare professionals in practice settings because they cannot be worn during certain clinical skills and may disrupt hygiene best practices. Although the study included a small sample size, it was intentionally conducted as a pilot investigation focused on assessing the feasibility and acceptability of wearable biosensors for monitoring biophysical indicators of stress and fatigue during simulation in which clinical skills are performed [52]. Future studies will measure multisensory physiological biomarkers among rural healthcare workers using a less intrusive, more hygienic, and operationally feasible biosensor, such as a wristband. Measurement of biophysical signals may inform the unique contributors to stress, as well as physical fatigue experienced by rural healthcare workers. To identify potential associations between burnout, physiologic stress responses, and physical fatigue among rural healthcare workers, participants will complete validated surveys on stress, physical exertion, and burnout before and after wearing the biosensor. Identifying associations between stress, physical fatigue patterns, and burnout risk leads to future predictive modeling for organizations to use for burnout risk prevention strategies.

#### 3.1.2. Burnout in Long-Term Care Nursing Assistants

LTC NAs encounter numerous challenges within their occupation, including staffing issues, burnout, and high turnover. NAs in U.S. LTC facilities are facing a critical staffing crisis, impacting staff well-being and resident care quality [32]. NAs experience stress within their role, including attributes of formal caregiver burden [54]. Burnout occurs frequently within the NA population [55]. Rural LTC NAs can face this stress and burnout, as well as traumatic events [56]. Researchers at this university completed a hermeneutic phenomenological study that explored the experience of NAs with traumatic events in the rural LTC workplace [56]. Interviews were completed with nine NAs who told of their traumatic experiences. The participants’ strength and compassion, yet vulnerability and a specific need for support, emerged from the interviews [56]. Within the workplace, traumatic events and burnout can be associated with turnover and care quality [57,58,59]. Future research is ongoing to better understand what NAs experience and to develop methods to address burnout, turnover, and care quality.

### 3.2. Workplace Violence

The Occupational Safety and Health Administration (OSHA) [60] describes workplace violence (WPV) as acts or threats of violence, including physical violence, harassment, and other threatening behaviors occurring in the workplace. Working with people who may be volatile or unstable or working in isolated areas have been identified as factors that increase the risk of violence for some workers [60]. Ongoing research continues to highlight the negative impact WPV has on the nursing workforce. In general, across both urban and rural environments, those working in healthcare settings are more likely to experience WPV compared to other workers and are five times more likely to suffer an injury related to workplace violence when compared to workers overall [61]. According to the National Database on Nursing Quality Indicators, two nurses are assaulted every hour [62]. The impact of WPV on nurses, combined with the staggering occurrence rates, is motivating research and regulatory change.

Violence towards nursing staff can come from an array of aggressors. In 2001, the Injury Prevention Research Center at the University of Iowa developed a typology of workplace violence through a panel of national experts that includes four types based on the aggressor–victim relationship:

- Type I—Criminal Intent
- Type II—Customer/Client
- Type III—Work-on-Worker
- Type IV—Personal Relationship

Classifying WPV into distinct types helps differentiate its underlying causes and inform targeted solutions. Type II violence, which is perpetrated by patients and/or visitors, is the most common type of workplace violence for healthcare facilities [63]. Meanwhile, Type III violence—often described as lateral violence, incivility, or bullying, where the aggressor is a fellow staff member—remains widespread across healthcare settings and continues to be researched heavily [64]. Even with the available literature, additional research and collaboration is needed [65]. Delaney et al. [66] identified five overlooked challenges in addressing workplace violence—including competing ethical demands, lack of proactive engagement, and systemic barriers—which suggests that a single evidence-based intervention protocol to address Type II violence remains unobtainable. Current CON research is being conducted through the following avenues: exploring the lived experience of rural ED nurses dealing with Type II violence, evaluating post-incident debrief tools, and examining Type III lateral violence and incivility.

#### 3.2.1. Workplace Violence Type II—Rural Nurse Experience with WPV

Emergency departments (EDs) see violent patients, and yet, unlike clinics that can administratively dismiss these patients, the ED must screen all patients seeking care according to the Emergency Medical Treatment and Labor Act (EMTALA). Less is known about WPV in rural healthcare settings, in part because it is thought to be underreported due to factors of caring for a rural population, including nurses who often know patients, as they are family or friends, and the close networking of smaller communities, which may lead to additional violence or threats if the behavior is reported [67]. There is some overlap in urban and rural areas as to why WPV is underreported, including the belief that violence is an expected occupational hazard and inconsistent data collection [68,69,70]. As a result, important questions remain regarding the unique experiences, risk factors, and workforce implications of WPV in rural healthcare settings [71].

Two faculty members at this university completed a descriptive, phenomenological study to learn more about WPV, specifically the experiences of nurses in rural EDs in SD. A total of ten registered nurses, all employed in Critical Access Hospitals across SD, participated in this study [72].

The five themes that emerged were: isolation, familiarity with patients, reliance on law enforcement, preparation or lack thereof, and chemical influence on patients. Two themes, isolation and familiarity with patients, were unique to nurses practicing in a rural setting. While security staff are common in larger urban facilities, it was found that participants in this study did not have security staff within their facility. Rather, nurses had a heavy reliance on law enforcement, which is an interconnected theme. However, some participants expressed that law enforcement was not always available when needed, as most rural areas have a small number of law enforcement officers monitoring a large geographical area. Participants also noted that, at times, law enforcement officers are on call at night, meaning the officer must be called in to respond to violence in the ED [72].

Familiarity with patients was another common theme expressed by participants. Participants identified this familiarity as both positive and negative. Participants recognized the positive in being familiar with a patient, as this could potentially defuse the situation. However, participants also recognized that being familiar with a patient often led to rationalizing the behavior (i.e., the patient is not always violent) or hesitancy to involve law enforcement [72].

This study added to the literature by describing rural ED nurses’ experiences with WPV. Given the qualitative design of this study, along with a small sample size, these findings are descriptive in nature and may not be generalizable to the WPV experience among all rural ED nurses. Recommendations based on this work include continuing to raise awareness about WPV and developing reporting tools. Additionally, the need for enhanced training to prepare and debrief after a violent event was identified [72].

#### 3.2.2. Workplace Violence—NAs

NAs practicing in LTC face barriers in addressing WPV, including appropriate ways to address injuries, violence, and lack of training. NAs have a high rate of injury and illness, often related to their physically demanding job [27]. NAs, including those in the rural setting, face WPV; however, the culture of addressing WPV in specific LTC settings can include acceptance with minimal administrative support [56,73]. NAs have described specific traumatic events they face in the rural LTC setting, including verbal or physical abuse from residents [55]. Rural LTC NAs have expressed a lack of appropriate training for difficult resident behaviors, dementia, and violence [56,73].

#### 3.2.3. Workplace Violence Solution—Post-Incident Debrief Strategies

Violence still occurs, and nursing unit managers struggle with effective post-incident actions even though post-incident debriefing is an organizational responsibility per OSHA guidelines and Joint Commission Standards [74]. Structured post-incident debrief protocols have been demonstrated to boost recovery and rebuild a sense of safety [66]. Yet significant literature gaps exist to guide post-incident care [74]. Considering many rural facilities already face staffing constraints and a shortage of seasoned staff, having experienced staff to provide feedback and mentorship post-violence encounters is even more scarce. To address this gap, the CON—through a PhD student’s dissertation efforts—is developing and evaluating self-reflection as a debriefing tool for use after violent encounters. Using a researcher-developed screen-based virtual aggression simulation, nursing students experience a violent encounter unfolding through sequential scenes and submit their considerations and responses to each video scene. Following the simulation, the experimental group of students self-rates their de-escalation performance using the English-Modified De-escalation of Aggressive Behavior tool. A final review of the simulation scenes allows all the students to adjust their scene responses. In a quasi-experimental design, students’ pre-test and post-test Clinical Confidence in Coping with Aggression results are collected. Study results are anticipated to be published in the summer of 2027. Statistically significant findings would encourage continued development of a self-reflection tool for nurse leaders and educators to facilitate an evidence-based post-incident learning and growth process and boost staff’s feeling of confidence in addressing future incidents.

#### 3.2.4. Workplace Violence Solution—Nursing Education

WPV solutions are not designed to have the nursing staff stray from their purpose but rather to provide them with the skills and knowledge to respond effectively to these incidents to maintain the core mission of nursing [66]. Training on health and safety is one of OSHA’s five core [75] WPV prevention components and ensures staff are aware of the WPV risks, understand how to protect themselves, and reduce the likelihood of WPV occurrences and injury.

Nursing students in the clinical setting are new to the hospital environment and culture, feel powerless, and are more likely to tolerate violence from patients [76]. Combined with the trend of nursing units being overwhelmed and a subsequent lack of support for students, nursing students are at an increased risk of suffering from WPV and require support, training, and oversight. A literature review on WPV training for nursing students found that this investment in didactic, role-playing, or simulation training pays off [77]. Positive results were seen in quantitative outcomes such as awareness, skill acquisition, self-efficacy, and confidence, as well as qualitative acceptance of the training. The authors encouraged continued research and development in this area but also published a mandate that WPV training be incorporated in all undergraduate and graduate schools of nursing to promote the safety and well-being of these students.

The CON continues to prioritize preparing nursing students for healthcare settings by providing WPV education in their curriculum and evaluating the effectiveness of this training. The core curriculum includes content on WPV. The virtual aggression simulation within the debrief studies above is being utilized to educate students on de-escalation practices.

#### 3.2.5. Workplace Violence Solution—NAs

Solutions to the challenges that rural LTC NAs face are multi-faceted. Rural LTC NAs are passionate about their roles, despite voicing a lack of support within their workplace [55]. This is promising because it indicates NAs want to work in their occupation, which is caring for residents. While there is not an extensive amount of research on rural LTC NAs, many solutions proposed in urban settings can be applied to the rural setting.

Solutions can be aimed at recruitment and retention and include training, resources, compensation, and mental health support. To address staffing issues related to recruitment, recommended interventions include appropriate marketing, networking, and stipends [32]. Further, to enhance retention, peer support programs, personal resiliency training, workplace culture training, and professional development enhancement should be implemented [32]. Collaboration amongst LTC facilities, colleges and universities, and other education programs can allow for adequate training. However, this may require additional funding through grants or other resources [78]. In addition, training NAs within facilities, as opposed to training through a program outside of the organization, may result in a higher NA retention rate, specifically with novice NAs [79].

Offering financial incentives, as well as providing increased compensation and adequate benefits, can reduce workforce shortages [78]. Further, to address compensation, policy changes related to Medicaid reimbursement should be considered, as the facility itself is often limited by outside regulations that then affect NA pay [32].

Mental health support and the prioritization of well-being and health are essential in reducing turnover and ultimately, ensuring quality resident care [32]. Self-compassion training has demonstrated a positive change in nursing home NAs’ skills in coping with stressors such as increased workload, decreased staffing and support from coworkers, resident behaviors, and feeling as though they are not valued team members [80]. Mindfulness-based interventions may also be beneficial for LTC NAs’ mental health [81]. Rural LTC NAs have suggested grief circles or debriefing interventions to assist with the aftermath of traumatic events that occur in rural LTC [56].

NAs experience many occupational challenges, yet there is little research specifically aimed at identifying rural LTC NAs’ occupational challenges and solutions. Ultimately, more research needs to be conducted with rural LTC NAs to identify additional methods of support for this unique population, focusing on staffing, coping, education, and management support [56].

### 3.3. Workplace Incivility

Workplace incivility (Type III workplace violence) is not a new concept. However, since the COVID-19 pandemic, there has been heightened awareness of this issue, especially in healthcare. According to Andersson & Pearson [82], workplace incivility is defined as deviant behavior that is low intensity with an ambiguous intent to harm. Lack of respect for others, eye-rolling, withholding information, and being discourteous or rude are examples of incivility and violate traditional workplace norms [82,83]. Incivility impacts not only the nurse or healthcare worker experiencing incivility but also negatively affects the organization and patient safety. Nurses may opt to leave their job or the workforce, leading to a loss of experienced nurses and potential short-staffing [84].

#### Workplace Incivility—Rural/Urban Comparison

While it is known that workplace incivility is a problem in healthcare, less is known about the relationship among workplace incivility, resilience, and the healthcare environment in rural and urban settings. Three faculty members at SDSU sought to determine if differences in incivility existed between rural and urban healthcare settings. Additionally, faculty sought to better understand the impact of incivility on job satisfaction for nurses, nurses’ intent to leave, and quality of patient care and safety [85]. This study utilized a descriptive correlational design using survey data collected from RNs, LPNs, and advanced practice registered nurses (APRNs) with an SD nursing license. The survey included two instruments: the Short-Negative Acts Questionnaire (SNAQ) and the Connor-Davidson Resilience Scale (CD-RISC), which measured workplace incivility and nurse resilience, respectively. In addition, demographic information, including highest degree earned, years of nursing experience, geographic location, intent to leave nursing position, and quality of nursing care, were also collected [85]. Surveys were completed by 625 participants, with 342 participants completing the survey in its entirety. A chi-squared test revealed no significant differences between the total survey population and the population used in the regression model.

The sample was overwhelmingly female (90.1%) and white/Caucasian (91.2%). The majority were employed full-time (81.9%) and in an urban setting (51.2%). Based on the mean SNAQ (17.4, SD 8.04) and CD-RISC scores (29.23, SD 6.19), there was a significant negative correlation between workplace incivility and resilience (*p* = 0.001). Workplace incivility was higher for rural nurses (M = 18.09) than for urban nurses (M = 16.74). However, this was not statistically significant (*p* = 0.12). Nurses with graduate degrees in nursing, while not statistically significant, had higher levels of incivility (M = 18.15, Sd = 9.219) and resilience (M = 30.28, SD = 6.824). These findings contribute to understanding the relationship between workplace incivility and resilience among nurses practicing in both rural and urban settings and highlight workplace incivility as an important issue affecting the nursing workforce [85].

This study added to the literature on the relationship between workplace incivility and resilience in rural and urban areas in one Midwestern state. The findings of this study suggest that workplace incivility remains an important concern across healthcare settings, both rural and urban, and warrants continued efforts to address uncivil behaviors that can threaten quality and safety for staff and patients [85].

### 3.4. Environmental and Occupational Health Challenges—Summary

Environmental and occupational health challenges in rural settings include healthcare workplace violence and incivility. While workplace incivility is not unique to rural healthcare, rural settings may experience different responses and impacts. By providing intentional education on workplace violence and incivility for nursing students and listening to rural nurses’ and CNAs’ voices, innovative strategies to address these concerns in rural healthcare can be developed. The CON is investigating strategies to not only impact the concerns of the rural healthcare workforce but also the health of rural populations.

## 4. Unique Populations Within the Rural Population

Nursing care in rural settings requires understanding the unique healthcare needs, resources, and challenges these communities face. This extends beyond caring for individual patients but focuses on addressing the needs of entire communities and populations, emphasizing culturally sensitive and community-driven solutions [86]. SD’s rural population includes culturally distinct groups such as Hutterite colonies, whose lifestyle and cultural traditions may influence health behaviors and healthcare utilization. Beyond cultural considerations, rural nurses must also address population health priorities as determined by the population, such as reducing barriers to oral healthcare access and improving care for children and youth with special health care needs (CYSHCN) [87]. These examples are provided so other colleges of nursing may replicate these efforts to improve rural health in their areas.

### 4.1. Hutterite Colonies

The CON has utilized population-based clinical experiences with Hutterite colonies to provide nursing students with a unique opportunity to explore the intersection of culture, community, and healthcare. For many students, this is their first exposure to the Hutterite way of life, which differs significantly from the mainstream U.S. population. Hutterite communities are organized around shared religious beliefs and cultural traditions that often influence health practices, family roles, and decision-making processes [88]. Daily life is centered around faith, family, and shared responsibility, with most colonies operating as self-sustaining agricultural communities. Students gain valuable insight into how culture, religion, social structure, and community values influence health beliefs, healthcare decisions, and ultimately health outcomes (E. Hofer, personal communication, 1 March 2025).

These experiences allow students to develop cultural awareness while recognizing the importance of providing healthcare in a manner that is respectful of cultural values and traditions. Students also gain exposure to a more patriarchal social structure, which challenges students to consider how family dynamics, decision-making processes, and communication patterns may influence healthcare delivery. This firsthand experience helps students better understand the importance of tailoring healthcare interventions to meet the needs of diverse populations.

During their population-based clinical rotations, nursing students collaborate with colony members and key stakeholders to identify health priorities and develop meaningful, population-focused interventions. Rather than imposing predetermined solutions, students work alongside community members to assess needs, identify resources, and create culturally appropriate health promotion strategies. For example, colony leaders and community members identified health topics of interest and worked with nursing students to develop educational initiatives, including first aid training, healthy meal preparation, and other health promotion activities tailored to the specific needs and priorities of each Hutterite colony.

This collaborative approach emphasizes partnership, respect, and community engagement while allowing students to apply public health, health promotion, and evidence-based practice principles learned in nursing school.

Projects developed by nursing students have addressed a variety of health concerns relevant to colony populations. Examples include oral health education programs, healthy nutrition and dietary initiatives, first aid training, cardiopulmonary resuscitation (CPR) education, injury prevention, and chronic disease prevention efforts. Through these projects, students learn to assess population health needs, design educational interventions, and evaluate outcomes while considering the community’s unique cultural context. These experiences strengthen students’ communication, collaboration, leadership, and health education skills while preparing them to provide culturally responsive care across diverse settings.

Ultimately, these clinical experiences on Hutterite colonies offer students a transformative learning opportunity that extends beyond traditional clinical settings. By interacting directly with a unique cultural community to improve health and well-being, students have an opportunity to develop a deeper appreciation for population health, SDOH, and culturally competent nursing care. These experiences reinforce the nurse’s role as both a healthcare provider and a community partner, preparing graduates to deliver effective, respectful, and holistic care to diverse populations throughout their professional careers.

### 4.2. Children with Oral Health Needs

An example of how the CON can help meet the needs of children and youth is a partnership between a large provider of dental benefits and insurance and the CON undergraduate nursing programs. Faculty secured funding to train nursing students in population health clinicals to work with underserved populations to identify specific population-level needs and provide focused health education, oral health assessments, and fluoride to consented individuals. Most of the grant work has focused on children, especially those without access to oral health resources or dental care not covered by Medicaid. Medicaid is a joint federal and state program offering healthcare coverage to individuals with limited income and resources in the U.S. [89]. Many of the activities have occurred at income-based preschools (such as Head Start programs), public elementary schools, and other populations identified as at risk or underserved.

The grant has provided oral health supplies, fluoride, and dental referrals to those in need for the last 4 years. Children in these populations receive free oral health screenings, education and hygiene supplies, and those who have signed parental consent can receive free fluoride varnish application. To date, nearly 10,000 elementary students have received oral health screenings, education, and supplies. Nearly 5000 of those students had signed consents and participated in free fluoride applications. Nearly 300 nursing students have been trained in oral health education and fluoride application and have provided oral health education and supplies to students in Head Start and elementary school children in public schools, as well as children from Hutterite Colonies. This information comes from the number of students enrolled at each agency and the number of returned consent forms allowing for fluoride participation. Relevant outcomes have included reports from Head Start and elementary school nurses of improved oral health practices among participating children. In addition, 11 students identified through nursing-led oral health screenings as requiring dental referral subsequently received donated dental services through these student-driven oral health programs.

This grant funding has driven ongoing research projects spanning all three sites of the CON to investigate the nature of oral health disparities faced by rural dwellers in SD. The research project has two arms: one focusing on barriers to dental care (cost, travel, access) and the other on nursing students’ knowledge and confidence in providing oral health education and assessment. Beyond meeting the needs of at-risk populations, this knowledge and skill set positions nursing students to recognize individual population needs and leverage their nursing education to gather resources and work with local agencies to creatively meet those needs.

### 4.3. Children and Youth with Special Healthcare Needs

Children and Youth with Special Healthcare Needs (CYSHCN) comprise a growing population of children who require services beyond those typically needed by their peers due to chronic physical, developmental, behavioral, or emotional conditions [90]. Approximately 14.1 million U.S. children, or 20% of those under age 18, meet this definition [90,91]. Advances in early diagnosis, treatment, and chronic disease management have improved survival and shifted attention toward long-term health, quality of life, and family well-being for CYSHCN [91,92]. Despite decades of efforts to promote the medical home model, which emphasizes coordinated, comprehensive, family-centered care [93], many CYSHCN continue to experience fragmented healthcare systems that place considerable coordination burdens on families [91]. Rural families face additional challenges related to provider shortages, geographic distance, transportation barriers, and limited specialty services, making coordinated care particularly difficult to achieve [94,95,96,97]. In SD, more than half of CYSHCN do not have a medical home, and over 40% lack needed care coordination, highlighting persistent gaps in care delivery [98]. In rural settings, the challenge is not simply defining the components of a medical home but understanding how to implement and sustain coordinated care within complex geographic, workforce, and organizational contexts.

Addressing these challenges requires more than expanding services; it demands stronger connections among healthcare, public health, educational, and community systems. Faculty members from SDSU are leading a community-based participatory research (CBPR) initiative to address gaps in care coordination for CYSHCN in rural communities. CBPR provides a framework for engaging families, healthcare professionals, public health agencies, and community organizations as equal partners in identifying barriers, setting priorities, and developing solutions [99,100]. This collaborative model is especially important in rural communities, where local context, workforce limitations, and existing community relationships shape care delivery [99]. In partnership with a statewide advisory committee comprising public health professionals, healthcare clinicians, and experts in the medical home model, this project seeks to understand barriers and facilitators to the medical home model and coordination of care for CYSHCN across SD. Over the next year, qualitative interviews and focus groups with key stakeholders will explore common challenges and opportunities within existing systems of care. The findings will inform the development of community-driven, systems-level strategies to strengthen the medical home and improve care coordination for rural children and families.

### 4.4. Unique Populations—Summary

Addressing health challenges in rural communities requires approaches that respond to the population’s unique cultural, social, and priority needs. Through community partnerships, service initiatives, and unique clinical opportunities, the CON contributes to efforts aimed at improving health outcomes, reducing disparities, and preparing nurses for the rural workforce. These efforts emphasize culturally responsive care, population health, and community engagement in supporting the development of sustainable solutions to complex rural health challenges.

## 5. Discussion

As SD’s land-grant university, SDSU is continually leading the way to address SD community needs, which may improve rural healthcare and health through a variety of education, partnerships, and research strategies. SDSU is carrying out the mission and responsibility of land-grant institutions in the U.S., set forth in the Morrill Act in 1862 [10], and the CON is leveraging the position of nursing as the most trusted profession in healthcare [8] to implement, evaluate, and sustain rural nursing workforce strategies. Additionally, the CON is focused on generating new knowledge related to healthcare workforce well-being to hopefully mitigate burnout and retain the workforce, and on priority challenges identified by communities within SD.

Through several HRSA-funded programs and the NANEC, educating nursing students for rural healthcare practice has been a long-standing and successful priority. Gaining experience in caring for individuals in rural settings has led many of these nursing students to work in a rural healthcare facility after graduation. This is consistent with research performed in medical schools, where exposure to rural practice does foster graduates choosing to work in rural areas [101]. Thus, it shows how important it is to provide experiential learning opportunities for students to understand what it is like to work in these rural areas, improving recruitment to rural practice. This cannot be accomplished solely by schools or colleges of nursing; rather, partnerships are imperative.

The CON has been building impactful partnerships with rural healthcare facilities across SD and the region. These partnerships provide students with clinical experiences in several different areas of nursing. Furthermore, the partnerships with rural healthcare facilities have grown over the last 4 years, and students are getting more opportunities to explore rural nursing in an area where they would like to work after graduation. In addition, the expansion of the online program will lead to many students having the opportunity to complete their education and clinical experiences close to home, thus relieving barriers and stressors they may encounter. This is imperative, as the strongest predictor of medical students choosing to practice in a rural area was growing up in a rural area [101]. When nursing students have these opportunities, it allows them to experience the benefits and unique challenges one encounters when working in a rural healthcare setting. While this report describes initiatives intended to strengthen the rural nursing workforce, educational experiences alone cannot explain rural employment outcomes. Individual career preferences, prior rural exposure, economic incentives, and regional workforce demand may also contribute to graduates’ employment decisions.

Addressing the needs of the future and current healthcare workforce in the rural setting is essential to ensure adequate care is provided to the rural population, whether it is in long-term care, outpatient settings, the community itself, or within critical access hospitals. By researching and then identifying supportive interventions against workplace violence and burnout, there will be a greater emphasis on rural healthcare workers’ well-being. This will positively impact the quality of care that healthcare workers are able to provide to the rural community. It has been shown that physicians with high levels of burnout are 50% more likely to make a medical error [102]. Furthermore, improving the well-being of our healthcare workforce will aid in the recruitment and retention of the workforce [102], highlighting how research can affect the healthcare workforce supply.

Education related to SDOH has also been completed by all nursing students attending this university. This information provides foundational knowledge for the students to help them understand the social aspects that impact patients and their ability to access and receive healthcare services [103]. The CON is currently working on ways to disseminate this information to all healthcare professionals across the state of SD to develop a healthcare workforce that is versed in SDOH and able to create systemic strategies to reduce social barriers to healthcare and improve patient outcomes [103].

The SDOH encountered in these settings are experienced by healthcare professionals as well. This can be found in many forms, including the lack of mental health care due to numerous barriers [104], which they may seek due to challenges in rural healthcare, such as professional isolation [7]. Additionally, they may see it in the lack of care available for their children, as childcare deserts are more common in rural than urban areas [105]. These SDOH may keep individuals who grew up in rural areas from earning a nursing degree and practicing in the communities they know so well.

SDSU CON is paving a road of access to higher education for rural residents in SD by fostering relationships with rural healthcare facilities so nursing students can complete their clinicals close to home. These strategies address the SDOH of our students, who are more likely to practice as a nurse in their rural community, as seen in the medical profession [101]. These students have personal and professional experience with SDOH, living in rural areas, and with rural healthcare challenges. This makes them ideal, trusted individuals to lead community-driven solutions that address rural health challenges and improve access to safe, quality care close to home [106].

Additionally, the healthcare needs of unique populations within rural communities highlight the importance of not only addressing workforce shortages and access challenges alone but engaging in partnerships that support the specific cultural, social, and system-level challenges that may influence health outcomes and create barriers to care. Sustainable improvements in rural healthcare require approaches developed through meaningful partnerships with communities and responsive to locally identified needs. Key features of successful community-engaged rural health initiatives include identifying and responding to community needs, providing services of value, community leadership and innovation, reputation and trust, consistency, and knowledge sharing and program adaptation [107].

### 5.1. Limitations

This experience-based institutional case report has several limitations. First, the approaches used to improve rural health care were implemented at a single institution with a heterogeneous population, which may not apply to other academic, healthcare, and rural workforce settings. Some analyses of the initiatives and research included incomplete survey responses, small sample sizes, and reliance on institutional data sources, which may have introduced response and reporting bias. The initiatives described were program-level, and the reported outcomes cannot be interpreted as true causal effects on rural health outcomes. In addition, some of the initiatives have incomplete program outcomes due to ongoing implementation of the initiatives and incomplete follow-up. Lastly, selection bias is inherent in many of the research studies conducted by the CON faculty due to convenience sampling.

### 5.2. Recommendations

Nursing education is at the forefront of combatting the rural healthcare crisis. Universities, especially land-grant universities, need to act and seek partnerships with rural communities and healthcare entities to address rural health challenges, including the nursing workforce shortage in these areas. Finding ways to provide opportunities and encourage students to experience rural healthcare, as well as bringing higher education to rural residents, is essential to improving rural health.

Most rural healthcare workforce studies have focused on physicians and lack evidence of other variables affecting the students’ decision to practice in rural versus urban areas. While exposing students to rural practice during their clinical experiences is a favorable strategy, more rigorous research is needed to fully evaluate this strategy and identify other factors that influence the students’ decisions [108,109]. A recent scoping review found that this gap in the rural healthcare workforce research remains, continuing to report a lack of evidence to fully evaluate the success of strategies [110]. Alarmingly, even less research exists on the rural nursing workforce and strategies for recruiting and retaining rural nurses [111,112]. While SDSU has worked hard to become a leader and increase the nursing workforce in rural areas, there is still much that needs to be done. Rural training opportunities need to not only continue but be expanded so students can participate in more training hours in rural communities. It will be imperative to conduct rigorous research on this strategy and others to fully understand the factors that influence students’ decisions to practice in rural or urban areas.

Education surrounding SDOH should be expanded and offered in a variety of ways throughout students’ education [113]. Regardless of where a student works, they are likely to encounter a patient with an SDOH-related barrier or stressor that may affect them. Furthermore, rural healthcare professionals may experience and be affected by the SDOH in the area where they live and work. This may also affect their health and well-being. Interventions and research on healthcare professionals’ health and well-being should be informed by the SDOH [114].

Rural healthcare professionals face distinct factors that impact their well-being, stress, and burnout. However, little empirical evidence supports interventions for the rural healthcare workforce. Future research must be rigorous to properly analyze what supportive interventions are most impactful [44].

Work is needed to ensure a safe working environment for individuals across all areas of healthcare in both rural and urban settings to keep the workforce strong. With the nursing shortage expected to continue over the next several years, future nurses need more thorough education to equip them with the skills to handle WPV challenges when they arise. Students need to be prepared to work in any environment, and rural nursing should be no different. OSHA notes that healthcare organizations should provide comprehensive workplace violence education that includes prevention strategies, warning-sign identification, response protocols, and clear communication that all reported incidents will be investigated promptly and followed up appropriately [60]. The authors encourage continued research and development in this area but also standardized WPV training requirements across states, as there is currently inconsistency, and many states have no requirements [115]. Furthermore, WPV training is another prime example of how a college of nursing can partner with healthcare facilities to promote the safety and well-being of the future and current healthcare workforce [116].

## 6. Conclusions

The SDSU CON experience illustrates the potential impact of a comprehensive, rural-focused approach to nursing education, workforce development, research, and community partnership. By aligning innovative educational delivery models with targeted workforce initiatives and community-engaged research, rural colleges of nursing can play a critical role in strengthening healthcare capacity, improving access to care, and advancing sustainable health outcomes in rural and frontier communities.

## Figures and Tables

**Table 1:** College of Nursing efforts to train nursing students in rural healthcare.

Program	Clinical Practice Area	Number of Participants	Principal Outcomes	Funding Source
NANEC * 2015–2026	Variety—same as all BSN students in the CON	66	83% retention 89% practicing in SD 55% practicing in Native American healthcare facility	SDSU CON Indian Health Services—scholarships
Rural Nurse Fellow * N/A–present	Rural 144 h preceptorship	100	N/A	CON
IMPACT-RNS 2018-present	Rural Primary 102 h	90	N/A	HRSA Donors
PREPARE-RNS 2022–2026	Rural Acute 80 h + 144 h preceptorship	33	94% completion >50% practicing in rural healthcare facility	HRSA
RETAIN-RNS 2025–2029	Rural Acute & Long-Term 124 h & 144 h preceptorship—acute 40 h—long-term	40	Pending completion of program	HRSA
GUIDE-RNS 2025–2029	Rural Acute & Long-Term 24 h clinical + 25.5 h simulation—acute 24 h clinical + 6.5 h simulation—long-term care	140	Pending completion of program	HRSA
Online BSN * 2026–present	Variety of Rural	96 (24 students annually)	Pending 1st cohort’s completion of program	CON
TOTAL		565		

Note. N/A: data is not available; all reported data is from program evaluation. * Program will continue beyond 2026.

## Data Availability

Two types of data are reported in this essay: (1) program evaluation data, which is not available to external entities, and (2) research data. For the latter, please refer to the original publication for data availability.

## Abbreviations

The following abbreviations are used in this manuscript:

CON	College of Nursing
DNP	Doctor of Nursing Practice
HRSA	Health Resources and Services Administration
RUCA	Rural–Urban Commuting Area
SDOH	Social Determinants of Health
LPN	Licensed Practical Nurse
LTC	Long-Term Care
RN	Registered Nurse
BSN	Bachelor of Science in Nursing
NA	Nursing Assistant
CNA	Certified Nursing Assistant
ADN	Associate Degree in Nursing
IMPACT-RNS	Impacting Models of Practice and Clinical Training for Registered Nurses and Students
NANEC	Native American Nursing Education Center
PREPARE-RNS	Partnering to Address the Critical Nursing Shortage in South Dakota
RETAIN-RNS	Partnering to Expand and Retain SD Nursing Workforce in Acute and Long- term Settings
GUIDE-RNS	Growing Underserved Communities through Innovation, Development, and Education for Registered Nurses and Students
NCPD	Nursing Continuing Professional Development
HRV	Heart Rate Variability
HR	Heart Rate
EDA	Electrodermal Activity
ECG	Electrocardiogram
GSR	Galvanic Skin Response
EMG	Electromyography
EEG	Electroencephalogram
ED	Emergency Department
WPV	Workplace Violence
OSHA	Occupational Safety and Health Administration
EMTALA	Emergency Medical Treatment and Labor Act
CYSHCN	Children and Youth with Special Healthcare Needs
CBPR	Community-Based Participatory Research
